# Correction: Winter habitats of bats in Texas

**DOI:** 10.1371/journal.pone.0221590

**Published:** 2019-08-19

**Authors:** Melissa B. Meierhofer, Joseph S. Johnson, Samantha J. Leivers, Brian L. Pierce, Jonah W. Evans, Michael L. Morrison

The fifth author's middle initial is incorrect. The correct name is: Jonah W. Evans. The correct citation is: Meierhofer MB, Johnson JS, Leivers SJ, Pierce BL, Evans JW, Morrison ML (2019) Winter habitats of bats in Texas. PLoS ONE 14(8): e0220839. https://doi.org/10.1371/journal.pone.0220839
